# Utilization of insecticide treated bed net and associated factors among households of Kola Diba town, North Gondar, Amhara region, Ethiopia

**DOI:** 10.1186/s13104-018-3697-7

**Published:** 2018-08-13

**Authors:** Melaku Birhanu Alemu, Meles Addisu Asnake, Melaku Yilma Lemma, Melkitu Fentie Melak, Melaku Kindie Yenit

**Affiliations:** 1 Department of Health System and Policy, Institute of Public Health, University of Gondar, Gondar, Ethiopia; 2 Deleta Chefa Health Center, Oromia National Regional State, Arsi, Ethiopia; 3 Sambo Bedala Health Center, Oromia National Regional State, Jimma, Ethiopia; 4 Department of Human Nutrition, Institute of Public Health, University of Gondar, Gondar, Ethiopia; 5 Department of Epidemiology and Biostatistics, Institute of Public Health, University of Gondar, Gondar, Ethiopia

**Keywords:** Utilization, Factors, ITN, Insecticide-treated bed net, Malaria, North Gondar, Kola Diba

## Abstract

**Objective:**

The primary aim of this study was to assess the utilization of an insecticide-treated bed net on the preceding night and associated factors among households of Kola Diba town in 2017.

**Results:**

Of the 260 households, 239 (91.9%) (95% CI = 88.5–95%) utilized insecticide-treated bed net on the night preceding the interview. 242 (93.1%) households didn’t treat the bed net whereas 18 (6.9%) treat the bed net by chemical regularly. Household structure and knowledge about malaria transmission were found to be associated with insecticide-treated bed net utilization.

**Electronic supplementary material:**

The online version of this article (10.1186/s13104-018-3697-7) contains supplementary material, which is available to authorized users.

## Introduction

According to WHO malaria report 2016, there were 212 million new cases of malaria and 429,000 estimated number of deaths worldwide in 2015; from this Africa accounts for 90% of malaria cases and 92% deaths [1]. Globally, there is 29% reduction in mortality rate associated with malaria because of the expansion of prevention and control measures in 2010. Sub Saharan Africa took the greatest portion of global malaria burden. There is around 75% of the territory and 60% of the population is at risk of malaria infection in Ethiopia [2, 3]. There are four main intervention strategies that are being applied in Ethiopia to combat malaria. These are early diagnosis and prompt treatment, selective vector control, the use of insecticide-treated mosquito nets (ITNs), and environmental management [4].

In the rural areas of the Ethiopia, there was a high distribution of ITN for free to decrease the burden of malaria as soon as possible. Because of that bed new ownership has risen exponentially. Despite this fact, utilization at night was optimal, in some occasions people use the net for another purpose or do not use them at all [5].

A community-based cross-sectional study conducted in Wonago woreda, Southern Ethiopia, showed that out of 944 freely supplied ITNs to 638 households, 649 (68.8%) were reported to have been used by the houses [6].

In a community-based cross-sectional study conducted on pregnant women in eight malaria endemic kebeles in northern Ethiopia, of the 815 households surveyed, 59% owned at least one non-long lasting or long-lasting ITN (59.5% rural vs. 54.5% urban) [7].

A cross-sectional study conducted in Uganda showed that prevalence of ITN utilization among children seeking health care was 34.2%. ITN utilization was higher among children aged < 5 years compared to children aged ≥ 5 years [8].

A community-based cross-sectional study conducted in Wonago woreda, Southern Ethiopia, reviled that; absence of separate bedrooms, low perception of ITN as the basic preventive strategy of malaria, and lack of sufficient ITNs supply affects ITNs utilization [7]. The utilization of ITNs has been found to vary with seasons of the year and the acceptability of the nets in terms of size, color, and shape. Demographic characteristics like age, education, size of household, and ethnicity influenced the use of bed nets [9]. A baseline interview in Mali revealed that the most common reasons for not treating nets were cost, availability, and lack of knowledge regarding the effectiveness of ITNs in preventing malaria [10]. A cross sectional investigation conducted in Uganda showed that ITN utilization was affected by the number of children below 12 years of age, source of mosquito nets, sharing beds with caregivers, and formal employment of heads of household [8].

Even though, there are some studies depicting ITN utilization and factors associated with it in Ethiopia, there was no study done in Kola Diba town, which is one of the most malaria prevalent area in Ethiopia. The aim of this study was to determine ITN utilization and associated factors among households of Kola Diba town in 2017.

## Main text

### Methods

A community-based cross-sectional study was carried out to determine the use insecticide-treated bed net, and associated factors. The study was conducted from February 8–12, 2017. Kola Diba town is found in North Gondar zone, Amhara region, 729 km North of Addis Ababa. The study population was households in kebele 01 and 02 Kola Diba. The heads of houses or any family members aged ≥ 18 years and lived for more than 6 months in the selected kebeles were included, whereas respondents critically ill, unable to communicate and had no bed nets were excluded.

The sample size was determined using Epi info version 7 statistical software and the total number of households (3552). The utilization of ITNs based on the history of sleeping under a net on the previous night was 76.8% in a previously done study in eastern Ethiopia [11] with a 5% margin of error. Finally, the sample (255) by adding 5% non-response rate the final sample was 268. Systematic random sampling was used with the sampling fraction Kth will calculated (k = N/n = 3552/268 = 13). Out of the first 13 households, the initial one was determined by using the lottery method. Utilization of insecticide-treated net is the dependent variable, whereas factors affecting the utilization such as affordability, knowledge, attitude, and practice were the independent variables.

An interviewer-administered structured questioner was first prepared in English, and then translated into the local language (Amharic), and then back-translated to English by investigators to maintain consistency. The data collection process was strictly monitored on daily basis by the supervisor (Additional file 1).

Data were entered by using SPSS (Statistical Package for Social Science) version 20 for analysis. Factors were tested using the bi-variable analysis, and a value ≤ 0.2 was a candidate for the multivariate analysis. The multivariable logistic regression analysis was done to identify confounders. Adjusted odds ratio (AOR) with a 95% confidence interval and p-value < 0.05 was used to show the association between explanatory and dependent variables. Variables with p-value of < 0.05 were considered as significant.

### Result

#### Sociodemographic characteristics

Out of the total 260 respondents, with 97% response rate, 93 (35.8%) of the responders were male and 64 (24.6%) respondents were housewives. Most of the respondent’s religion was orthodox accounts for 203 (78. 1%). Most of the respondent’s age group was between 18 and 25 with the mean age of 37.45 years (Table 1).

**Table 1:** Sociodemographic characteristics of respondents in Kola Diba, 2017

Variable	Frequency (n)	Percentage (%)
Age		
18–25	63	24.2
26–45	126	48.5
> 45	71	27.3
Sex		
Female	167	64.2
Male	93	35.8
Occupation		
Housewife	64	24.6
Civil servant	60	23.1
Student	48	18.5
Farmer	41	15.8
Merchant	35	13.4
Other	12	4.6
Family size		
1–3	48	18.5
4–5	89	34.2
> 5	123	47.3
Religion		
Orthodox	205	78.8
Muslim	55	21.2

#### Intra-household dynamics

##### Household structure

Most of the houses were made of mud which accounts for 236 (90. 8%). Most of the household has two rooms which account about 101 (38%). The majority of the respondents had two beds in their house which accounts 136 (52.3%) and only 22 (8.5%) percent had greater than three beds within the household. 60 (23.1%) households have only one bed (Additional file 2).

##### Decision making, prioritization and seasonal variation of bed utilization

Out of 260 the households, 154 (59.2%) said that fathers determined who should sleep under the nets. Children and pregnant women in the household are given highest prioritization for bed net use which accounts 215 (82.7%) and 153 (58.8%) respectively. Most (95.4%) and the list (63.8%) bed net use was reported during summer and winter respectively (Additional file 3).

##### Utilization of bed net

Out of the 260 respondents, 239 (91.9%) (95% CI 88.5–95%) were found to sleep under bed net in the night preceding the day data were collected.

#### Knowledge

Out of the 260 respondents, 218 (83.8%) had good knowledge about the transmission of malaria, and 178 (68.5%) knew about its prevention.

The level of satisfaction of using bed net in the prevention of malaria is 155 (63%) responders that bed net is very effective in the prevention of malaria 87 (35.4%) responders that bed net is effective in the prevention of malaria.

Of the 242 (93.1%) respondents who didn’t treat their bed nets, whereas 18 (6.9%) treated them by chemicals regularly. Out of 242 who didn’t treat the nets with chemicals, 125 (51.4%) lacked knowledge followed by lack of knowledge accounts for 114 (43.8%) and other causes are accounts for 4 (1.6%). Almost all of the respondents 258 (99.2) they received the nets from government, and 255 (98.1%) responders got the net for free (Additional file 4).

From 18 respondents who treat bed net by insecticide chemical 14 (77.8%) use treating station. 2 (14.3%) took greater than 30 min to reach to the site (Table 2).

**Table 2:** Site, time and price to treat bed net with chemicals of ITN in Kola Diba, 2017

Variable	Frequency (n)	Percentage (%)
Site to treat		
Treat it myself	4	22.2
Treat at station	14	77.8
Time to reach site (min)		
< 10	7	50
10–20	1	7.1
20–30	4	28.6
> 30	2	14.3
Price to treat		
Free	14	77.8
< 5 ETB	2	11.1
5–15 ETB	2	11.1

*ETB* Ethiopian Birr, *min* minute

#### Factors affecting utilization of insecticide treated bed nets

Households who have Houses made from cement is 97.7% times less odd of using insecticide-treated bed net than households made from mud. Households who do not know about transmission of malaria is 90.8% times less odd of using insecticide-treated bed net than who know about transmission (Table 3).

**Table 3:** Multivariable logistic analysis of factors associated with ITN utilization

Variable	Utilization of ITN in the previous night		COR	AOR
	Yes	No		
Household structure				
Made of mud	222	18	1	1
Made of cement	17	3	2.176 (0.583, 8.131)	0.023 (0.002, 0.291)
Knowledge about transmission				
Yes	217	16	1	1
No	22	5	3.082 (1.030, 9.221)	0.092 (0.15, 0.563)

*COR* crude odds ratio, *AOR* adjusted odds ratio

p < 0.05 in multivariable logistic regression is significant

### Discussion

In this study, among the 260 net-owner households, the utilization of ITN by household members was 91.9% (95% CI 88.5–95%) the night preceding resumption of the study. Our finding is higher not only than those of studies conducted in Arbaminchi [12] and eastern Ethiopia w [13] which was 73% and 21.5% respectively, but also higher than investigations conducted on under-five [14], Federal ministry of health [15] and WHO target objective [16] which are 58%, 60%, and 80% respectively.

Several studies suggest that perceived malaria risk and malaria knowledge are important determinants of bed net ownership and utilization [17–19]. Among the 260 respondents 230 (88.5%) have knowledge about the transmission of malaria of whom 228 (99.1%) know that malaria is transmitted by the bite of a female anopheles mosquito suggesting a higher awareness compared to a study conducted in South-central Ethiopia and reported that 63% of the respondents perceived malaria as a disease transmitted by mosquito bites [20].

Household structure and knowledge about the transmission have a high association with the utilization of insecticide-treated bed nets in this study which in line with a study conducted in Ghana, where knowledge of malaria transmission was reported to be strong determinant of ITN usage [21, 22].

Out of the potential associated factors explored regarding the utilization of ITNs among households in Kola Diba, sufficient knowledge about transmission of malaria and ITNs, and household structure are found to be significant. Respondents who do not knew about malaria transmission have 0.092 times less odds of using insecticide-treated bed net than those who knew [AOR = 0.092 (0.15, 0.563)]. This is because if people know about malaria infection and the transmission methods, they use ITN to protect themselves. This is also supported by studies conducted in Bahir Dar, north-west Ethiopia [23].

In conclusion, there is a higher utilization of ITN in Kola Diba compared to the objectives set by FMOH in 2007. This is because of high knowledge about the prevention and transmission of malaria and the high availability of ITN in the households.

## Limitation

When interpreting the findings of this study, scholars need to take into consideration the limitation that the cross-sectional nature of the study design made it impossible to arrive at the causal relationship between the different explanatory variables.

## Additional files

**Additional file 1.** Questioner.

**Additional file 2: Table S1.** Household and bed net characteristics of the respondents in Kola Diba.

**Additional file 3: Table S2.** Decision making, prioritization and seasonal variation in bed net utilization in Kola Diba.

**Additional file 4: Table S3.** Source and price of bed nets.

## Abbreviations

WHO: World Health Organization
ITNs: insecticide treated nets
LLINs: long-lasting insecticide-treated nets
RBM: roll back malaria
IRS: indoor residual spraying
MIS: Malaria Indicator Survey
SPSS: Statistical Package for Social Science
AOR: adjusted odds ratio
