# Supplementary material for: Utilization of insecticide treated bed net and associated factors among households of Kola Diba town, North Gondar, Amhara region, Ethiopia
Source: BMC Res Notes. 2018 Aug 13;11:575. doi: 10.1186/s13104-018-3697-7 (PMC6090723; doi:10.1186/s13104-018-3697-7)
Supplement: Supplementary file 1 — Additional file 1. Questioner. [file 13104_2018_3697_MOESM1_ESM.pdf]

Questioner

**Part 1**	**Sociodemographic characteristics**	
1	Interviewee relation to the household head	1. Household head (Mother, Father…)<br>2. child<br>3. servant<br>4. others, specify------------------------------
2	Age	
3	Sex	
4	Occupation	
5	Family size	
6	Religion	

**Part 2**	**ITN utilization in the household**		
1	What is your housing structure?	1. Hut<br>2. Made of mud<br>3. Made of cement<br>4. Other -----------	
2	How many rooms does your house have?	____________________	
3	Does all family members have a bed?	1. Yes<br>2. No	
4	If yes to Q3, how many beds are there?		
5	Do you use bed nets in this family?	1. Yes<br>2. No	*If no, skip to #15*
6	Does all beds have bed net?	1. Yes<br>2. No	
7	Do you use the bed net when you go to sleep?	1. Yes<br>2. No	
8	Do you cover yourself well?	1. Yes<br>2. No	
9	How money bed nets are there in this family?	1. Over 3<br>2. 3<br>3. 2<br>4. 1	
10	Who decides whom to sleep under bed nets?	1. Father<br>2. Mother<br>3. Other, specify ……………	

11	If there are no enough bed nets, to whom do you give priority?	1. For children under 5<br>2. For pregnant mother<br>3. For lactating mother<br>4. For head of households<br>5. Other, specify-------------	
13	In which season doses the family use bed nets more frequently?	1. Spring<br>2. Summer<br>3. Autumn<br>4. Winter	
14	Do you use bed net in the previous night?	1. Yes<br>2. No	
15	If your answer is no, why don't you use the bed net?	1. Doesn't protect from malaria<br>2. Creates high temperature<br>3. Causes skin irritation<br>4. It takes time to make the bed net<br>5. The mosquito may not bite us<br>6. Other, specify---------------------	
**Part 3**	**Knowledge, Attitude and Perception**		
1	Do you know ways of transmission of malaria?	1. Yes<br>2. No	*If no, skip to #3*
2	If yes to Q1, what are they?	…………………………………………<br>…………………………………………	
3	Do you know prevention method of malaria?	…………………………………………<br>………………………………………….	*If no, skip to #5*
4	If your answer is yes, what are they?	…………………………………………<br>…………………………………………	
5	Do you know insecticide treated bed nets?	1. Yes<br>2. No	
6	Do you have chemically treated bed net in the households?	1. Yes<br>2. No	*If no, skip to #11*
7	Do you know the difference between chemically treated and untreated bed nets?	1. Yes<br>2. No	
8	If yes, what is the difference?	……………………………………………<br>…………………………………………….	
9	Where did you get the information about the difference between chemically treated and untreated bed nets?	1. Radio<br>2. Health professionals<br>3. Friends<br>4. Pastor<br>5. TV/video<br>6. Newspaper<br>7. Others...........	

10	What is your opinion about the used of chemically treated bed net and malaria prevention	1. Very effective<br>2. Effective<br>3. Neutral<br>4. Less effective<br>5. Ineffective	
11	Why didn't you use chemically treated bed net	………………………………………<br>………………………………………	
12	Do you treat the bed net with chemicals?	1. Yes<br>2. No	
13	Do you know how often the bed nets must be chemically treated?	1. Yes<br>2. No	
14	If yes, how often?	…………………	
15	Do you think treated bed nets may useful for malaria prevention?	1. Yes<br>2. No	
16	If yes, explain why?	…………………………………<br>…………………………………	
17	If no, list reasons?	…………………………………	
**Part 4**	**ITN Availability**		
1	Is there a place where you can get bed nets?	1. Yes<br>2. No	
2	Where did you get the bed nets you are using now in the family?	1. Government<br>2. NGOS<br>3. Pharmacy<br>4. Supermarket<br>5. Mini market<br>6. Kiosk<br>7.	
3	How much does one bed net costs?	………………………… ETB	
4	How does the bed net distribute to the household?	1. By number of family<br>2. By number of rooms<br>3. By number of beds<br>4. Other, specify----------------	
5	Have you ever treated your bed net chemically?	1. Yes<br>2. No	
6	If you don't, why?	……………………………………………<br>……………………………………………	
7	If yes, when is the last time you treated your bed net?	1. 1 year ago<br>2. 6 month ago<br>3. 3 month ago<br>4. Every month	

		5. -----------------	
8	Do you treat the bed net by yourself or you go to treating site?	1. By myself<br>2. treating sight	
9	Do you know the bed net chemical treating site?	1. Yes<br>2. No	
10	How much time it takes to do to treating sight?	10 minute<br>10-20 minute<br>20-30 minute<br>over 30 minute	
11	How much it cost to treat the bed net with chemical?	………………………ET. birr	
