# Supplementary material for: Utilization of insecticide treated bed net and associated factors among households of Kola Diba town, North Gondar, Amhara region, Ethiopia
Source: BMC Res Notes. 2018 Aug 13;11:575. doi: 10.1186/s13104-018-3697-7 (PMC6090723; doi:10.1186/s13104-018-3697-7)
Supplement: Supplementary file 2 — Additional file 2: Table S1. Household and bed net characteristics of the respondents in Kola Diba. [file 13104_2018_3697_MOESM2_ESM.pdf]

Table S1: Household and bed net characteristics of the respondents in Kola Diba, 2017

Variable		Frequency (n)	Percent (%)
**Housing structure**	Made of mud and hut	240	92.3
	Made of cement	20	7.7
**Number of room**	1	45	17.3
	2	101	38.8
	3	83	31.9
	>3	31	11.9
**Number of bed**	1	36	13.8
	2	136	52.3
	3	66	25.4
	>3	22	8.5
**Number of bed net in the household**	1	60	23.1
	2	109	41.9
	3	84	32.3
	>3	7	2.7
