# Supplementary material for: Utilization of insecticide treated bed net and associated factors among households of Kola Diba town, North Gondar, Amhara region, Ethiopia
Source: BMC Res Notes. 2018 Aug 13;11:575. doi: 10.1186/s13104-018-3697-7 (PMC6090723; doi:10.1186/s13104-018-3697-7)
Supplement: Supplementary file 3 — Additional file 3: Table S2. Decision making, prioritization and seasonal variation in bed net utilization in Kola Diba. [file 13104_2018_3697_MOESM3_ESM.pdf]

Table S2: Decision making, prioritization and seasonal variation in bednet utilization in Kola Diba, 2017

**Variables**		**Decision making and prioritization in using bed net**	
		Yes	No
**Decision maker**	Father	154(59.2%)	106(40.8%)
	mother	138(53.1%)	122(46.9%)
**Priority for bed net use**	Children	215(82.7%)	45(17.3%)
	Lactating mother	25(9.6%)	235(90.4%)
	Pregnant mother	153(58.8%)	107(41.2%)
	Head of household	11(4.2%)	249(95.8%)
**Season**	Summer	248(95.4%)	3(1.2%)
	Autumn	178(68.5%)	82(31.5%)
	Winter	165(63.5%)	95(36.5%)
	spring	166(63.8%)	94(8.1%)
