# Supplementary material for: Utilization of insecticide treated bed net and associated factors among households of Kola Diba town, North Gondar, Amhara region, Ethiopia
Source: BMC Res Notes. 2018 Aug 13;11:575. doi: 10.1186/s13104-018-3697-7 (PMC6090723; doi:10.1186/s13104-018-3697-7)
Supplement: Supplementary file 4 — Additional file 4: Table S3. Source and price of bed nets. [file 13104_2018_3697_MOESM4_ESM.pdf]

Table S3: Source and price of bed net

| variable | | Frequency (n) | Percent (%) |
|---|---|---|---|
| **Source of ITN** | Government | 258 | 99.2 |
| | Super market | 1 | 0.4 |
| | Other | 1 | 0.4 |
| **Price of ITN** | Free | 255 | 98.1 |
| | <50 birr | 5 | 1.9 |
| **Ways of ITN distribution** | Family size | 198 | 76.2 |
| | Number of rooms | 19 | 7.3 |
| | Number of beds | 34 | 13.1 |
| | Other | 9 | 3.5 |
| **total** | | 260 | 100 |
